# The feasibility and acceptability of delivering a group trauma‐focused intervention to children in care

**DOI:** 10.1111/bjc.12494

**Published:** 2024-07-25

**Authors:** Rebecca S. Davis, John Devaney, Sarah L. Halligan, Richard Meiser‐Stedman, Paula Oliveira, Patrick Smith, Paul Stallard, Rebecca Kandiyali, Alice Phillips, Aalia John, Rachel M. Hiller

**Affiliations:** ^1^ Department of Psychology University of Bath Bath UK; ^2^ School of Social and Political Sciences University of Edinburgh Edinburgh UK; ^3^ Department of Clinical Psychology and Psychological Therapies University of East Anglia Norwich UK; ^4^ Anna Freud National Centre for Children and Families London UK; ^5^ Institute of Psychiatry, Psychology and Neuroscience King's College London London UK; ^6^ Department of Health University of Bath Bath UK; ^7^ Warwick Medical School University of Warwick Coventry UK; ^8^ Division of Psychology and Language Sciences University College London London UK

**Keywords:** complex trauma, feasibility, foster care, group treatment, PTSD

## Abstract

**Objective:**

Young people in care (i.e., in the child welfare system) are a group who have often experienced very high rates of potentially traumatic events, including maltreatment. It is well‐documented that they have high rates of trauma‐related mental health difficulties, such as posttraumatic stress. To address the needs of the large number of young people who may benefit from support, scalable interventions are crucial. But also important is that they are effective and deliverable – particularly given the complexity of this group and services. We assessed a five‐session group CBT‐based intervention for PTSD. The primary goal was to understand core procedural and protocol uncertainties to address prior to a definitive trial.

**Methods:**

Participants were 34 10–17 year olds in care, with moderate to severe posttraumatic stress symptoms, and their caregiver. We ran seven groups (four online), delivered in social care and NHS‐based mental health teams. Data were collected via pre‐, post‐, 3‐month follow‐up questionnaires and qualitative interviews.

**Results:**

Of the 34 participants allocated to the intervention, 27 (80%) attended at least three of the five sessions (most attended all). Caregiver attendance was lower (50%). There was generally good completion of assessment measures. Qualitatively, most participants were positive about the intervention, and many reported improvements in areas such as coping, sleep, and willingness to talk about experiences. However, there were important concerns about the lack of ongoing support, given this was a low‐intensity intervention for a group who often had complex needs.

**Conclusion:**

The intervention and research protocols were acceptable to most young people and carers. With modifications, a future definitive trial would likely be possible. However, key considerations include: how (and whether) to screen for PTSD; the trial design; and the option to embed high‐intensity support (e.g., via assessing a stepped‐care model).

Young people entering the care system (i.e., care of the child welfare system/State/local authority; formal terminology differs by country) often have histories of exposure to significant trauma and adversity (Department for Education, 2022). The most common reason for being moved into the care system is abuse and neglect, with exposure to domestic violence, parental mental health difficulties, and drug and alcohol abuse all common (Department for Education, 2022). Once in care, these young people remain at increased risk of future trauma exposure and exploitation compared to their peers (Shaw & Greenhow, 2020). There is well‐documented evidence of the effect of these experiences on the mental health of this group of young people. Approximately 50% of young people in care meet criteria for at least one diagnosable mental health condition, with complex comorbidities common (Bronsard et al., 2016; Ford et al., 2007; Lewis et al., 2019). Many also likely experience elevated sub‐clinical symptoms that still have a substantial impact on their wellbeing. The unaddressed mental health needs of children and teens in care has been identified as one of the key drivers of poor outcomes that can occur over the lifespan, including high rates of school exclusion, homelessness, unemployment, and adult mental health difficulties (Jones & Morris, 2012; Teyhan et al., 2018).

One mental health outcome affecting many young people in care is posttraumatic stress disorder (PTSD). PTSD is a trauma specific mental health condition, with rates 12 times higher than in non‐care‐experienced peers (Ford et al., 2007). Symptoms include re‐experiencing (e.g., nightmares, flashbacks), avoidance (e.g., of talking or thinking about the trauma(s)), altered arousal (e.g., difficulty concentrating, sleeping), and negative cognition and mood (e.g., thoughts like ‘I cannot trust anyone’, shame, fear, low mood) (American Psychological Association, 2022). Cognitive models of PTSD highlight various processes that can lead to and maintain the sense of ongoing danger and threat inherent in PTSD. This includes maladaptive cognitive appraisals, disjointed or disorganized trauma memories, and avoidant coping, which interact to drive the development and maintenance of symptoms (Ehlers & Clark, 2000). Such models have received wide empirical support (e.g., Gómez de La Cuesta et al., 2019; Mitchell et al., 2017; Trickey et al., 2012) including in longitudinal work specifically with young people in care (Hiller, Meiser‐Stedman, et al., 2021). For this group of young people, maladaptive cognitive appraisals and avoidant coping were particularly robust drivers of PTSD, as well as the more recently proposed complex PTSD (Hiller, Meiser‐Stedman, et al., 2021; World Health Organization, 2019).

The first‐line recommended treatment for young people with PTSD, including following complex trauma or maltreatment, is a trauma‐focused cognitive behaviour therapy (tf‐CBT) (National Institute for Health and Care Excellence [NICE], 2018). This is usually delivered as a 1:1 intervention over 8–12 sessions, with more sessions (e.g., 20+) often needed for young people with more complex presentations (Cohen et al., 2012). There is extensive evidence for the effectiveness of tf‐CBTs, including for children exposed to more complex trauma (Bennett et al., 2021; Cohen et al., 2012; Mavranezouli et al., 2020; Sachser et al., 2017). However, the high‐intensity nature of the intervention, combined with the well‐documented capacity issues within both child and adolescent mental health services (CAMHS) and children's social care, and the high number of young people in need (Children's Commissioner for England, 2023; NHS Confederation, 2022), means this intervention alone (or indeed any high‐intensity intervention) is unlikely to feasibly meet the needs of the large numbers of young people in care who could benefit from support. Understanding whether lower‐intensity options may be useful in this context, for this group of young people, is an important area of investigation.

One option to reach a larger number of young people in need is a group approach. Group approaches not only allow more young people to receive a treatment, they often require less specialist training than high‐intensity approaches (e.g., trauma‐focused CBT, EMDR), and are designed for delivery by a range of people and professionals, including non‐mental health experts (e.g., teachers) and trained community members, such as community leaders (Davis et al., 2023). Our recent meta‐analytic review found that group CBT‐based trauma‐focused interventions were effective at reducing PTSD symptoms in young people (Davis et al., 2023). Although individual psychotherapy was found to be superior (and hence the recommended approach where possible), compared to no treatment at all (i.e., passive control) or even other treatments (i.e., active control), group CBT‐based treatments led to moderate reductions in PTSD symptom severity, as well as reductions in depression symptoms.

One such group‐based approach is Teaching Recovery Techniques (TRT) developed by the Children and War Foundation (Yule et al., 2013). This five‐session programme (with two additional sessions for caregivers) uses CBT‐based strategies to target trauma‐related distress. There is good emerging evidence, including from a meta‐analytic review, that this intervention significantly reduces trauma‐related distress (Davis et al., 2023). However, much of the evidence base is from samples of children exposed to war, natural disaster or community violence (e.g., Barron et al., 2016, 2021; Chen et al., 2014; Pityaratstian et al., 2015). There has been little research using these approaches with samples of young people in care. A small study of 17 young people in a secure unit setting in the UK found some initial evidence that TRT was effective at reducing distress (Barron et al., 2017).

As a first step in understanding whether TRT may effectively address the trauma‐related distress of young people in care, we conducted a feasibility and acceptability study. The primary aim was to assess the feasibility and acceptability of using TRT with young people in care with elevated PTSD symptoms, delivered across a social care and specialist mental health service. We were interested both in the appropriateness of procedural decisions (e.g., the feasibility of conducting a next‐step powered randomized controlled trial (RCT); of screening for PTSD at social‐care level, fidelity to the manual) and general acceptability of the intervention.

## METHOD

### Governance and pre‐registration

Ethical approval was provided by the United Kingdom (UK) Health Research Authority (Ref 20/WA/0100) and University, with further approvals from the host trust and local authority. The trial was pre‐registered on ClinicalTrials.gov (NCT04467320) and the protocol published in *Pilot and Feasibility Studies* (Hiller, Davis, et al., 2021).

### Design and protocol changes

The study was originally designed as a feasibility and pilot RCT, with TRT delivered in‐person, starting in 2020. As outlined in the protocol paper (Hiller, Davis, et al., 2021), the goal was to randomize 50 young people in care, aged 10–17 years old, with 25 ultimately being offered the intervention and 25 in a care‐as‐usual (CAU) arm. However, due to challenges with screening rates (described in detail in Results and Discussion), the randomized component was abandoned (a key feasibility question) and the trial became an open feasibility pilot, with all young people being offered TRT. Due to the COVID‐19 pandemic the intervention was also tested both as an online and as an in‐person intervention.

Potentially eligible young people were screened for PTSD symptoms using the Child Revised Impact of Events Scale (CRIES‐8) screening tool, delivered either via their social worker, mental health worker or foster carer. To participate, informed consent was required by relevant local authority staff (e.g., social worker, team manager), with informed assent from the young person (or consent if 16+ years), and informed consent for the caregivers' own participation. Following consent, young people and their primary caregiver or keyworker (if the young person lives in a residential care home a keyworker is a named staff member who has responsibility for the child) completed assessments at baseline, post‐intervention and 3‐month follow‐up.

Originally, to be included in the trial, the young person was required to score 17 or above on the CRIES‐8 (the cut‐off for clinically‐elevated PTSD symptoms; Perrin et al., 2005). However, in discussion with the independent steering committee this was changed to scores of 14 or above, reflecting moderate to severe PTSD symptoms. That change partly reflected service concerns that young people were scoring just below 17 but still experiencing major functional impairment related to their symptoms. This decision was also informed by evidence that young people in care tend to under‐report their symptoms (Tarren‐Sweeney, 2019) and that sub‐syndromal PTSD can be equally as debilitating as PTSD meeting full diagnostic criteria (Zlotnick et al., 2004).

### Sample

Young people were recruited from a single urban local authority based in the South West of England. A small number of young people (*n* = 3; of whom 2 participated) came via a second small neighbouring local authority, who shared some mental health resources with the larger authority.

Eligible young people had to be under the care of a local authority but could be in any type of placement, with the exception of living with a biological parent. Inclusion criteria were being 10–17 years and under the care of a local authority, and experiencing elevated (moderate to severe) symptoms of PTSD. Exclusion criteria were severe active suicidal ideation, psychosis, moderate to severe learning disability (defined as being educated outside of mainstream learning due to learning difficulties), not being fluent in English, and currently receiving direct trauma‐focused therapy.

**FIGURE 1:**
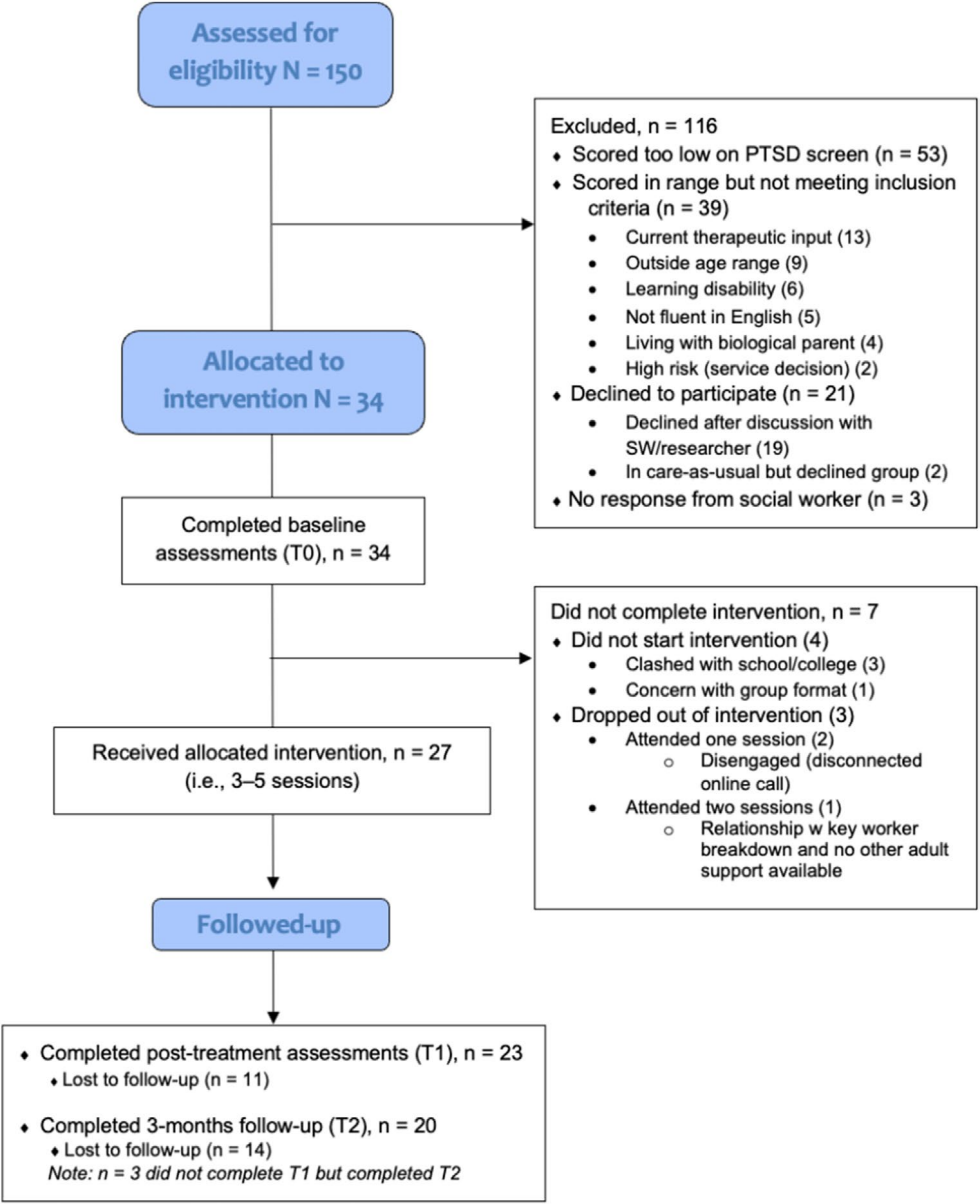
CONSORT flow diagram.

The final sample comprised 34 young people aged 10–17 years old, and their primary caregiver or keyworker. Demographics are presented in Table 2, with the Consort flow‐chart in Figure 1.

#### Screening

Young people were screened using the CRIES‐8 (Perrin et al., 2005). This tool was used because it is well‐validated in various trauma‐exposed population and, crucially, it is short (only 8‐items). Social work teams were trained in recognizing PTSD and using the CRIES‐8 screening tool and were asked to use the tool with young people on their caseload. Young people were eligible for the study is they scored 14 or higher on this measure, reflecting moderate to severe PTSD symptom severity.

### Intervention

Teaching Recovery Techniques (TRT) was delivered as a 7‐session group intervention. TRT was originally developed by the Children and War Foundation for children exposed to war, and has been extensively evaluated in this population (see Davis et al., 2023). We made some minor revisions to the manual for young people in care (e.g., updated case study, language). TRT is ultimately a skills‐teaching group, using CBT‐based techniques to manage symptoms, which are practiced in‐session. The intervention comprised 5‐sessions for the young person and 2‐sessions for their caregiver, with caregiver sessions coinciding with the earlier young person sessions. Sessions were weekly for 90 min and delivered by two trained mental health professionals. Young people were grouped by age, with a child group (approx. 10–13 years) and teen group (approx. 14–17 years); allowing for some clinical judgement as to whether a 13 or 14 year old might prefer the younger or older group. Following an intervention manual, session one provides psychoeducation and introduces a case example of a young person in care (which is referred to in multiple sessions); session two focuses on intrusive images, worries and dreams; session three on arousal, emotions and coping; session four on avoidance, memories and triggers; and session five on memories and ‘wrapping up’. The intervention does not involve in‐depth reliving or extensive trauma narrative work, as would be found in a 1:1 intervention. The two caregiver sessions coincided with the first two young person sessions and focused on psycho‐education, learning about what the young person would be learning and practicing, and supporting the young person through the intervention (see Yule et al., 2013).

Of note, although there are other evidence‐informed group‐based interventions for children and teens with PTSD (see Davis et al., 2023), we chose TRT for the following reasons: (i) TRT is only five‐sessions for young people, making it one of the quicker group interventions available; (ii) TRT has a good evidence‐base for effectively treating PTSD in children exposed to war, where many would have complex comorbidities and where there may be ongoing environmental risk or instability, which suggested it might offer a similarly suitable approach for young people in care; and (iii) TRT was developed by clinicians based in the UK, meaning training was readily accessible.

#### Therapist training

Therapists came from a range of mental health professional backgrounds, including drama and art therapists, psychotherapists and children's wellbeing practitioners. An initial group (*n* = 8) received four online half‐day training sessions in the TRT manual (equivalent of 2‐day training). Supervision was also provided by clinical psychologists during the intervention. Due to staff changes, new therapists also joined the intervention and were trained on the manual by those originally trained (i.e., using a ‘train the trainer’ model).

#### Fidelity checks

Therapist adherence to the treatment manual was explored through fidelity checks, including session observations by the research team and self‐completed checklists by the therapists (indicating whether or not they included key components and why). Therapists were also asked to audio record one session for each group. To do this, they were first required to seek permission from young people prior to the session so they could opt‐out, in which case the session would not be recorded. A key objective was to explore what types of fidelity checks were feasible.

### Measures and analysis

#### Qualitative outcomes

Feasibility and acceptability were primarily assessed via qualitative interviews with the young people and their caregiver. These were 1:1 semi‐structured interviews, either in‐person or via video call. Interviews were audio‐recorded and transcribed. They were analysed using reflexive thematic analysis. Two researchers analysed the interviews, and all coding was quality checked by RD, who also conducted the interviews (RD was not involved in intervention delivery). Interviews and focus groups were also run with the therapists who delivered the intervention. The full analysis of the therapist interviews is not included in the current paper, however key themes that overlap with caregiver and young person report are summarized.

#### Quantitative outcomes

Participating young people and carers completed questionnaires online, using Qualtrics. The primary quantitative outcome was the child self‐reported PTSD symptom severity on the Child and Adolescent Trauma Screen (CATS), a 20‐item questionnaire on DSM‐5 PTSD symptoms, rated on a scale of 0 (never) to 3 (almost always). Quantitative outcomes are listed in Table 1; for full details of these measures, see Hiller, Davis, et al. (2021). Of note, three PTSD‐focused tools were used in this pilot. The CRIES‐8 provided a short validated screener (which was then collected for consistency); the CATS (primary outcome) was added as a longer validated screening tool that assesses all core DSM‐defined symptoms (as this might be important for a future trial, if there was exploration of symptom cluster level change); the CPSS was used to assess for the presence and absence of a full diagnosis of PTSD, as this may also be useful for a future trial. Ultimately, as this was a feasibility study, the primary goal of these measures was to determine their acceptability and feasibility (e.g., rate of completion).

**TABLE 1:** Quantitative outcome measures collected pre‐ and post‐treatment and 3‐month follow‐up.

Measure	Construct	Reporter	
		YP	Carer
Child and adolescent trauma screen (CATS)^a^	PTSD symptoms	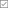	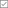
Child Revised Impact of Events Scale (CRIES‐8)	Initial screening instrument; PTSD symptoms	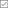	
Child PTSD Symptom Scale (CPSS)	PTSD diagnosis [present/absent] and symptom severity	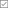	
Strengths and Difficulties Questionnaire (SDQ)	Total and externalizing difficulties	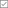	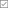
Short Mood and Feeling Questionnaire (SMFQ)	Depression symptoms	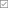	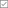
The Inventory of Parent and Peer Attachment (IPPA)	Attachment difficulties	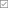	
The Parent Trauma Response Questionnaire – support subscale (PTRQ)	Caregiver support style		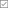
The Child Health Utility 9D (CHU‐9D)	Quality of life	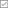	

Abbreviation: YP, young person.

^a^ Child‐reported CATS is the primary outcome, whereas the remaining instruments are secondary outcomes.

#### Quantitative symptom analysis

We were unable to follow protocol and test post‐treatment between‐group effect sizes (due to lack of randomization). However, for transparency we made an a priori decision to report effect sizes (Cohen's *d*; with 95% CI) for pre‐post change and pre‐follow‐up change. Using Cohen's *d* a small effect size would be *d =* 0.2, medium would be 0.5, and large would be over 0.8.

## RESULTS

### Descriptives

#### Participant characteristics

Participants were 34 10–17‐year‐olds (*M* = 13.4 SD = 2.3) under local authority care and their primary caregiver (or key worker if in residential care). The vast majority of young people were living in a non‐biological foster placement (*n* = 29), with three placed with a kinship carer and two in a residential setting. Table 2 presents detailed descriptive statistics. All young people were experiencing moderate to severe PTSD symptoms, based on the CRIES‐8 (mean score at screening = 25.3).

**TABLE 2:** Participant demographic characteristics.

	Children (*N* = 34)	Carers (*N* = 34)
Gender, *n* (%)		
Female	18 (53%)	33 (97%)
Male	15 (44%)	1 (3%)
Other	1 (3%)	0 (0%)
Age (years), mean (SD)| *n* (%)	13.4 (2.3)	
25–34 years old		3 (9%)
35–44 years old		4 (12%)
45–54 years old		9 (26%)
55–64 years old		7 (21%)
≥65 years old		3 (9%)
Missing		8 (23%)
Ethnicity		
White British	16 (47%)	18 (53%)
Mixed White and other	6 (18%)	0 (0%)
Other ethnicities	4 (12%)	8 (23.5%)
Missing	8 (23%)	8 (23.5%)
Length of time in care (years) missing *n* = 3	4.0 (2.7)	
Length of current placement (years), mean (SD) missing *n* = 9	3.1 (3.1)	–
Number of placements, *n* (%)		
1 placement	11 (32%)	–
2–3 placements	15 (44%)	
4–5 placements	4 (12%)	
9 placements	1 (3%)	
Missing	3 (9%)	

We received completed maltreatment checklists from 22 social workers (i.e., reports on 65% of the sample). On this checklist, they reported either confirmation or strong suspicion of different types of maltreatment: neglect (*n* = 20; 91%), emotional abuse (*n* = 17; 77%), exposure to domestic violence (*n* = 16; 73%), physical abuse (*n* = 13; 59%), and sexual abuse (*n* = 11; 50%).

#### Screening rates and conversion

Of the social workers involved, 83% (38 of 46) used the CRIES‐8 with at least one of their young people. In addition to social worker screening, nine young people were also screened from the waitlist of the linked specialist mental health service (children looked‐after CAMHS). In this case, for those who scored above the cut‐off, the research team contacted their social worker or team manager to seek consent. Approximately 13% of the sample was screened via their foster carers, who were contacted by the research team, on behalf of the social care site. The full flow of participants is presented in the Consort diagram (Figure 1).

Of all young people screened (*n* = 150), 65% (*n* = 97) scored above the symptom cut‐off (14+ on the CRIES‐8) for eligibility. Of those 97, 40% (*n* = 39) were ineligible for other reasons. Of the participants who were eligible and contactable (*n* = 55), 21 declined and 34 agreed to participate. This reflects relatively poor conversion from screening to participation (34 of 150; 23%), but moderate conversion from invitation to participation (34 of 55; 62%).

#### (Lack of) randomization

As discussed in the Methods, the trial was originally designed as a feasibility RCT (see Hiller, Davis, et al., 2021). Ultimately, after many months of effort, randomization was dropped to allow young people to access the intervention and test other important feasibility and acceptability question. This decision was made in collaboration with the services and the independent trial steering group. This means a key finding was that randomization into a group‐intervention trial was not feasible within a single children's social care site. This was largely driven by challenges in having social care staff screen young people, and relay contact details to the research team, at a rate that meant eligible young people could be randomized into a group.

### Intervention groups and treatment completion

We ran seven groups, four of which were with younger participants (age range 10–13 years) and three with adolescents (age range 14–17 years). Four groups were delivered online, one of which was the pilot cohort, and three groups were in‐person. The pilot intervention and protocols were identical, except young people completed a reduced questionnaire pack (specified in Table 3) and caregivers were not required to complete the questionnaires (meaning the total possible carer cohort was *N* = 30). The group sizes ranged from three to six young people. Of the 34 young people allocated to the intervention, 80% completed at least three of the five sessions: 23 (68%) completed all five sessions; four (12%) completed 3–4 sessions; three (9%) completed 1–2 sessions; and four did not complete any sessions (12%). Of the caregivers, 17 (50%) completed both caregiver sessions; seven (21%) completed one; and 10 (29%) completed none. Reasons given for those young people who ‘dropped out’ of the intervention before completing all five sessions, are presented in the Consort figure. Of note, although these are small numbers, there was no pattern where a particular demographic (e.g., age, gender) were more or less likely to disengage.

**TABLE 3:** Descriptive statistics for outcome measures.

Child report	T0: Pre‐intervention *M* (SD), range	T1: Post‐intervention *M* (SD), range	T2: 3‐month follow‐up *M* (SD), range
	*N* = 34	*N* = 23	*N* = 20
Primary outcome			
CATS (PTSD severity)	27.88 (13.77), 3–53	21.57 (12.94), 0–46	22.65 (15.01), 0–52
Secondary outcomes			
CRIES‐8 (PTSD severity)	22.12 (9.90), 1–40	20.35 (10.55), 0–38	17.96 (10.84), 0–38
SMFQ (depression)	10.48 (7.14), 0–24	8.70 (6.84), 0–22	8.55 (7.51), 0–24
CHU‐9^a^	.84 (.13), .52–1.00	.86 (.11), .57–1.00	.84 (.14), .57–1.00
IPPA^a^	115.60 (16.53), 81–140	117.96 (19.87), 80–140	112.58 (24.39), 65–140
SDQ total^a^	19.63 (5.16), 10–29	18.55 (4.01), 12–28	17.75 (5.32), 8–28
SDQ externalizing^a^	9.07 (2.84), 4–14	9.00 (2.62), 5–15	8.56 (2.61), 5–13
CPSS^a^, ^b^ (PTSD severity; interview)	19.64 (13.34), 2–61	12.68 (8.51), 3–29	16.62 (14.60), 2–50

Carer report^a^	*n* = 25	*n* = 22	*n* = 17
CATS (PTSD severity)	30.21 (14.76), 5–53	26.90 (15.41), 1–57	27.88 (14.05), 6–53
PTRQ (negative appraisals)	9.82 (3.52), 3–18	8.87 (4.08), 1–18	10.13 (2.99), 6–15
SMFQ (depression)	10.22 (6.55), 1–22	10.00 (6.02), 0–22	10.29 (5.60), 1–21
SDQ total	19.67 (4.41), 13–28	18.68 (4.77), 13–28	19.47 (5.61), 12–31
SDQ externalizing	8.79 (2.86), 4–14	8.27 (2.37), 4–13	8.82 (2.96), 5–14

*Note*: These are the scores for the total sample who completed the measure at each time point. Across all measures higher scores mean worse outcomes, with the exception of CHU‐9. The sample size is the overall sample who completed follow‐up assessments, but completion rates were slightly lower for some measures (IPPA was completed by 20 participants at T1 and 15 at T2; CHU‐9 was only fully completed (i.e., no items missed) for 14 at T1 and 15 at T2).

^a^ These instruments were not administered to the 4 participants in the pilot group.

^b^ The sample for this measure was smaller: 25, 19 and 13 for T0, T1 and T2, respectively.

### Treatment fidelity

Therapists completed fidelity checklists for 71% (25 of 35) of the sessions, showing generally high willingness and capacity to complete these checks. From the checklist data, self‐adherence to session guidelines was generally good, with 68% reporting that they followed >70% of session manual guidelines and delivered all core activities. Therapists also provided reports on session length, which ranged from 1 to 1.5 h. Reports of adaptations were frequent, but minor, and open‐report feedback suggests this was often adapting the pace to fit the age and concentration levels of the group (e.g., giving additional examples, providing more time for personal reflection and mutual support).

To test for fidelity to the treatment manual, we aimed to record one session for each group. Ultimately, we received three recordings from three different groups. In four cases, the therapists either forgot to record the session, the recorder did not work, or they were unable to check with the young people beforehand (the protocol ensured young people were asked *before* the group so they could opt‐out confidentially). For the three recorded sessions, there were no major adaptations noted, and the therapist completed all key parts of the session manual. Two sessions were online, which meant minor adaptations were noted around slide sharing and using an online whiteboard.

### Completion rates of outcome measures

The post‐treatment follow‐up was on average nine‐weeks following baseline, whereas the three‐month follow‐up was on average 24 weeks post‐baseline. All young people (*N* = 34) completed their baseline assessment. Of young people who completed baseline assessments (T0), 79% (27 of 34) completed at least one follow‐up assessment; 23 (68% of baseline) completed post‐treatment assessments (T1) and 20 (59% of baseline) completed their 3‐month follow‐up (T2; 3 of whom had been lost at T1). For eligible caregivers (*N* = 30), 23 completed baseline (77%), 22 completed post‐treatment (73%) and 16 (53%) completed 3‐month follow‐up. Overall, four caregivers provided no data at any time point, but of those who provided a baseline assessment only two did not provide any follow‐up data.

Of those who completed the follow‐up assessments, the primary outcome measure (CATS) was completed by all. The questionnaire pack was ordered by importance (i.e., with primary outcome measure first), and in general, later questionnaires were completed slightly less frequently (e.g., at 3‐mo all who completed the questionnaires completed the PTSD and depression symptom measures, but 75% (15 of 20) completed the IPPA (attachment) and CHU (health economics) measures). The CPSS (PTSD diagnostic interview), which required scheduling a visit or videocall with participants, was completed by fewer participants at all time points (see Table 3).

Of note, of the seven young people who did not begin the intervention or dropped out early, none completed any follow‐up assessments.

### Description of usual care

As this was originally planned as a feasibility RCT, we collected descriptive data to understand what ‘usual care’ was for this group. We asked all young people and their carers to report on where they had previously accessed mental health support, from a range of categories of potential support options. Only approximately one‐third (*n* = 13) of the participants had accessed support from a professional mental health service prior to this trial, based on young person or carer report. Approximately 80% endorsed going to social care for support with their mental health (e.g., talking to their social worker), whereas 59% had sought support via school. In general, where they had accessed mental health support differed widely and spanned all options given, including helplines, primary care, and the voluntary, social care, education, and health care sectors.

### Symptom change

See Table 3 for average scores across all measures and Table 4 for change scores for all measures. For the primary outcome, based on the effect size there was a small to moderate reduction in PTSD symptom severity.

**TABLE 4:** Mean change in symptom scores.

(*n*)	Pre‐ to post‐test		Pre‐ to 3‐month follow‐up	
	Mean difference (SE) 95% CI	Cohen's *d* 95% CI	Mean difference (SE) 95% CI	Cohen's *d* 95% CI
Child report				
CATS (23, 20)	5.48 (2.65) [−.02, 10.97]	.43 [−.001, .86]	5.80 (3.77) [−2.09, 13.69]	.34 [−.11, .79]
CRIES‐8 (23, 20)	1.57 (1.99) [−2.56, 5.69]	.16 [−.25, .57]	6.74 (2.64) [1.22, 12.27]	.57 [.09, 1.04]
SMFQ (23, 20)	.91 (1.36) [−1.90, 3.73]	.14 [−.27, .55]	1.65 (1.24) [−.95, 4.25]	.30 [−.16, .74]
CHU‐9 (14, 15)	−.009 (.04) [−.19, .48]	−.10 [−.56, .37]	−.03 (.04) [−.37, .13]	−.15 [−.68, .38]
IPPA (20, 15)	.64 (2.40) [−4.37, 5.66]	.06 [−.38, .50]	.75 (3.11) [−5.91, 7.41]	.06 [−.45, .57]
SDQ total (19, 16)	1.85 (.84) [.09, 3.61]	.49 [.02, .95]	3.06 (1.49) [−.11, 6.24]	.51 [−.02, 1.03]
SDQ externalizing (19, 16)	.50 (.60) [−.77, 1.77]	.19 [−.26, .63]	.88 (.89) [−1.02, 2.77]	.25 [−.26, .74]
CPSS (19, 13)	3.84 (1.83) [−.00, 7.69]	.48 [−.00, .95]	.00 (3.95) [−8.61, 8.61]	.00 [−.54, .54]
Carer report				
CATS (20, 14)	5.44 (3.08) [−1.00, 11.88]	.40 [−.07, .85]	.78 (2.67) [−4.98, 6.54]	.44 [−.10, .96]
PTRQ (20, 14)	.50 (.73) [−1.03, 2.03]	.15 [−.29, .59]	.34 (1.13) [−2.10, 2.78]	.08 [−.45, .60]
SMFQ (22, 16)	.32 (1.46) [−2.71, 3.35]	.05 [−.37, .46]	1.03 (1.64) [−2.47, 4.53]	.16 [−.34, .65]
SDQ total (21, 15)	.95 (.84) [−.81, 2.71]	.25 [−.19, .68]	.80 (1.16) [−1.69, 3.29]	.18 [−.34, .69]
SDQ externalizing (21, 15)	.29 (.50) [−.76, 1.34]	.12 [−.31, .55]	−.13 (.73) [−1.70, 1.43]	−.05 [−.54, .45]

*Note*: Values are only calculated for the subsample that has completed both time points; therefore, they differ from those presented on Table 3. For all measures, a score above zero reflects improvement.

Abbreviations: CI, confidence interval; SE, standard error.

### Qualitative findings: The views from young people, caregivers and therapists

Twenty‐two young people and 23 caregivers (including two key workers) completed post‐intervention qualitative interviews. All young people had completed between three and five sessions. All therapists who delivered the intervention, completed an interview.

### Theme 1: Benefits and challenges of delivery format

#### Pros and cons of online v in‐person delivery

Overall, for those who completed the intervention online, caregivers and young people reported that the format allowed an increased sense of safety. For some young people, it allowed a safe person to be present (i.e., their caregiver), whereas the in‐person groups were young person only. For some young people, it also allowed a sense of anonymity (e.g., through having cameras turned‐off). Some endorsed that they would not have attended a group in‐person. This was sometimes for practical reasons (e.g., caregiver would not have been able to transport young person), but sometimes it was about emotional safety.

Whenever it's face to face… I get quite shy… And I knew that it would be safer [because] you [foster carer] were next to me. (young person, 10yo)

It's an unfamiliar environment in person, you know the office. “Who's going to be in there?” “who's going to leave?”. All that sort of thing. So, I think that [sessions being online] gave us an advantage in engaging him eventually. (carer)

However, many caregivers and some young people reported that the online format hindered engagement and facilitated avoidance, potentially reducing any benefits of the intervention. In particular, caregivers reported that the online format allowed young people to disengage when content became more challenging.

…because he could just mute you or switch you off, or move the screen so you couldn't see him. (carer)

Well, sometimes I could hide away. (young person, 11yo)

…if there are questions you didn't want to answer, you can kind of go on or you could go “sorry, what was that I didn't hear you”. (young person, 15yo)

Both caregivers and young people felt that the online format of delivery hindered their ability to build rapport and connect with others, meaning they were less likely to share their thoughts and feelings. Young people reported that selective participation from others (such as having their camera off) led to feelings of disconnection with the group. In contrast, a central positive theme from many young people who participated in‐person, was a feeling of connection, safety, and relatability with their peers (discussed in Theme 3).

…it's up to them if they don't want to have that camera on… it can be feeling a little bit, just odd. It doesn't make it feel like you're chatting with someone, it just seems like you're chatting to a computer. (young person, 15yo)

I think the group was okay. If there was a group of other young people, you should tell them to put on their camera, all of them, so that it's more easy to talk and build confidence. (young person, 17yo)

#### Therapist views on delivery format

All therapists delivering the group expressed barriers and concerns associated with online delivery. Although they appreciated the benefits that young people, caregivers and themselves experienced from online delivery (including practical benefits from not having to travel to a specific location and it meaning certain young people felt more comfortable taking part), therapists all reported concerns about young person engagement online. For some groups, no, or very few, young people turned‐on their cameras and participation was minimal, making engagement and rapport building challenging. Therapists reported difficulty recognizing and dealing with issues such as disengagement, avoidance, and issues with understanding the content, as well as ensuring the group could bond, and general safeguarding. Most therapists also found the online format meant they were not able to offer the level of individual check‐in and support they would have wanted to.

With one of the boys, whose goal had been [redacted]…I thought that was really important that he was saying that, but what I would have wanted, we couldn't split off; I couldn't wander over to him and go, “how's it going”, just spend a minute or two with him just helping to move that to the next stage, so that was a bit that was missing really. (therapist)

Not knowing what is going on the other side of the camera for the young people, particularly with the camera off. You know they could be doing anything. (therapist)

### Theme 2: The role of carers

#### Supporting and reinforcing learning

Carers reported feeling more able to offer emotional support to their young person, as a result of engaging with the carer sessions and having an increased knowledge of the impact of trauma.

When you have been living with someone for a long time you almost forget, don't you? Which has been a good reset and reminder for me, to think about that actually, this may impact upon him. (carer)

Many carers who attended the carer sessions spoke about the positive impact of having learnt about the tools introduced through the intervention, and how this allowed them to support their young person to use these outside of the sessions.

I have been able to say to (young person name) you need to go and find that safe place, or you need to do the breathing, or do you need to share what you are worried about? (carer)

#### Carer engagement

A number of carers who did not attend the carer sessions explained that they were unable to do so because of other commitments, including work and childcare. Of those who did attend, a minority reported that the sessions did not cover anything ‘new’ or that they had not previously learnt in other training.

…not new but recapped on things I had done over the years. I have been a foster carer for a long time, so it's not going to be new. (carer)

A small number of carers reported seeing the intervention as something which was for the young person and consequently they sought less involvement.

It wasn't for me to know what happened. It wasn't for me to know what he was talking about. It's his safe space. (carer)

#### Therapist views on the role of the carer

The majority of therapists spoke about the importance of carers in engaging young people with the intervention and maximizing positive outcomes. Additionally, they felt that carers' knowledge of their young person could help to tailor the intervention to that young persons' specific difficulties.

I also did that session with carers present with the two younger ones and that, in some ways, was probably easier because the carers kind of did know something and were able to prod them into something they actually wanted to achieve, whether they were willing to disclose that or not. (therapist)

### Theme 3: Facilitators of positive change

Carers and young people reported a range of positive impacts including more control over intrusive memories, a decrease in anger, reduction in traumatic flashbacks and scary dreams, improved sleep, and an ability to calm themselves down more quickly.

Actually because of all of the tools that we've put into our toolbox, like I've been able to use them when I feel like I've wanted to hit out or something. I've been able to use them, calm myself down, and just get on with a normal day. (young person, 15yo)

#### Shared experiences

The majority of carers and young people, particularly those who participated in‐person, spoke about the positive impact of being with other young people with whom they had shared experiences, including early trauma, subsequent mental health difficulties, and the experience of being in care. These shared experiences led to young people feeling more able to talk about their past traumatic experiences openly, underpinned by feeling understood and less alone.

And just like, being away with other people that sort of, like are there for the same reason, it was nice to know that they were there for that as well and it wasn't just me. (young person, 12yo)

You can talk to a social worker, but I don't think they 100% understand what you've been through. So, talking to other kids who are in the same situation as you was quite nice, because you know, how they're going to react to it. (young person, 15yo)

You know, to be part of a group where you actually feel like you are not alone, and this is not happening to just you. (carer)

Carers also spoke about their own shared experiences and the importance of being able to connect and share learning with other carers.

So for me it was really nice to hear other people's stories. And just think, other women I respect and how they deal with things and their sense of humour, and their experiences and how they have tried different strategies. (carer)

#### Tools to support coping and emotional regulation

Although there were some caregivers who struggled to understand the tools learned, as their young person did not wish to share, for the majority of carers and young people who completed the intervention, across both delivery formats, they spoke about the tools and techniques learnt in the group that were helpful for supporting mental health needs. Many carers saw the intervention as offering a foundational set of tools which could be developed over time.

I think (young person name) got some good base support that hopefully he can build on over the years. (carer)

Tools such as ‘safe space’ and breathing exercises were regularly cited as helpful, and the practical, active nature of the tools was valued.

Usually, I get angry because the memories pop up in my head again, I get angry and blame stuff on people. But then [foster carer] helped me to use the safe place and I did. (young person, 11yo)

There are some things that changed in my life, I think when you do the breathing, and before you go to bed, you do breathing and that might bring sleep. (young person, 17yo)

#### Increased capacity to seek and offer support

Many carers shared that their young person was more open, as a result of engaging in the intervention, particularly regarding past traumatic experiences. For some, it was the first time that their young person had begun talking about their early experiences.

I think he's beginning to be able to talk much more now about what's going on for him, I think and that took a lot for him to open up and say things which he's beginning to do now, and I think he finds it easier now to talk about things. (carer).

Similarly, a number of young people reported feeling more confident to talk about their experiences and seek support from others, including their carer and friends.

I think that it helped you kind of learn about, that it's okay, you can speak about, and even though it is a hard thing that's happened to you, at the end of the day, there's people there who want to help you, they aren't there to judge. (young person, 15yo)

### Theme 4: Challenges and barriers

#### The impact of revisiting past traumas

A small number of young people shared that they found revisiting traumatic memories and experiences particularly challenging and felt ill equipped to manage the strong emotions that this brought up for them.

I didn't like imagining what's happened to you, changing it to black and white, turning up the volume, turning up the sound, changing it to colour, making it blurry, because after, it stays in your head, it doesn't go when you turn off that TV remote, it doesn't go with you. (young person, 15yo)

A minority of carers shared similar views, adding that some young people felt ‘blindsided’ by how directly the group addressed traumatic memories.

…it brings things up that he didn't want to talk about it. That bit of it was really difficult for him. (carer)

A small number of carers and young people also spoke about the young person seeking to avoid particular content or even entire sessions because of concerns about the emotional strain of reliving early life experiences.

…it was dragging out all these bad memories. It was like going back to stage one. And he was saying to you at some point, why do I have to go. (carer)

…one of our conversations, out of it, was “I don't want to do this”, and I said, “why not”, and he said “I've learnt how to push it all down. And I don't want it coming up”. (carer)

#### Increase in challenging behaviour

Linked to the revisiting of traumatic memories, some caregivers felt that the group had ‘opened a can of worms’ or ‘pandora's box’, and a small number spoke about increases in anger, sleep difficulties and more challenging behaviours, which they attributed to the group.

But I think school seen more of the behaviours than we did, so at the beginning (young person's name) was going to school after the meeting and then I think after two sessions then they asked us to keep (young person's name) home from school. (carer)

…when he was doing it, his sleep was even worse. It was even worse. It was literally a couple of hours. His bedroom was above ours, and I am a light sleeper. He was up and about. (carer)

A minority of carers felt that these potential challenges should be made clearer at the outset of the group and expressed how they had caused emotional strain.

…because obviously, if you really know potentially what you are heading towards you can prepare yourself. And it was, it was really quite upsetting to see him in such a state. (carer)

However, some of these carers did identify that this increase in difficulties did often get better over time.

…it gets worse before it gets better. And it's… it's a long process. You don't see results overnight. (carer)

#### Need for ongoing support

Many of the caregivers and some young people spoke about how they felt that five sessions was a helpful start but not enough, particularly within the context of having brought up complex traumatic memories and experiences.

I do think it could require a little longer. I do think like, it's a bit like opening pandoras box isn't it? It's just like, opening these things about emotions and stuff like that, and then it's just like five weeks is not really enough is it? (carer)

…it would have been nicer if we could go back again just to have to explore more things that could possibly help. (young person, 12yo)

Unfortunately, due to major capacity issues across mental health services, further evidence‐based support (e.g., trauma‐focused CBT) was rarely available.

…we have been desperately trying to get some help for (young person name) really for the last 18 months, since she came to us. (carer)

“And it actually bought home to me actually how serious this could get if it's not dealt with. And that is why I am pushing for other interventions and stuff so, um yes, I am just going to keep going with that. (carer)

## DISCUSSION

The primary aim of this study was to assess the feasibility and acceptability of using a low‐intensity group CBT‐based intervention (Teaching Recovery Techniques) with young people in care with elevated PTSD symptoms, when delivered across a social care and specialist mental health service. We sought to investigate core procedural and protocol uncertainties for a later‐stage definitive trial, as well as exploring the acceptability of the intervention and key practical considerations from the perspective of those involved, particularly young people and carers.

### Acceptability of the intervention

There were few exclusion criteria in this study to ensure participants reflected those young people who may usually present at mental health services. Many of the young people in the group were experiencing complex mental health difficulties beyond PTSD symptoms, and some were experiencing other risks or complexity (e.g., uncertainty about placement, self‐harm). Despite these complexities, based on qualitative feedback, the intervention was generally well‐received. Of those invited to participate, over half agreed, and where young people declined it was commonly because of practical issues (e.g., timetable clashes), or because they did not wish to engage in any mental health support (group based or otherwise). There was also some preliminary evidence of a small to moderate change in PTSD symptom severity on the primary screening tool (CATS) and the CRIES‐8. Although this was not replicated on the diagnostic tool (in terms of symptoms reported), this tool was completed at far lower rates (only 38% of young people completed this measure at the final follow‐up).

Further supporting acceptability, of those who began the intervention, the majority completed all sessions. Where young people initially agreed but ultimately did not complete the intervention, some were for practical reasons, but in some cases it was due to the content of the intervention. For the latter, in many of these cases the young person did not have a consistent caregiver who was able to support them through the intervention. In our recent meta‐analytic review of group treatments for PTSD, caregiver involvement in the intervention did not moderate treatment effects (Davis et al., 2023). However, caregiver involvement may not always equate to caregiver support (i.e., a caregiver may not be involved, but still support the young person through the intervention). In practice, young people in care in the UK can struggle to access mental health support because of a lack of placement or caregiver stability (Phillips et al., 2023). Blanket service rules around access based on placement stability or support networks are problematic, particularly given the well‐established associations between the mental health of young people in care and placement instability (e.g., Konijn et al., 2019). However, further work is needed to understand how best to support young people in care through trauma‐focused interventions, when there may not be a trusted or consistent adult for support.

Only 50% of caregivers attended both caregiver training sessions, despite them being online (as reportedly preferred, to increase accessibility). This is lower caregiver engagement than studies testing TRT with caregiver sessions in the post‐war context (e.g., El‐Khani et al., 2021). As reflected in the wider literature, it might be that many foster carers already felt overcommitted (e.g., Hannah & Woolgar, 2018) and did not feel they had capacity to attend the sessions. From our qualitative feedback, some thought it was not necessary as the treatment was for the child. Understanding how best to support foster carers in these interventions is crucial, particularly given the mixed evidence for the impact of caregiver‐focused components on child outcomes (e.g., Davis et al., 2023; El‐Khani et al., 2021) and evidence from a small RCT that specifically tested a foster carer engagement component alongside gold‐standard trauma‐focused CBT and found it did decrease drop‐out (although had no impact on treatment satisfaction or children's clinical outcomes; Dorsey et al., 2014).

Although some carers were positive about the content, some also reported that it was similar to previous trainings and wanted more intensive focus on the practicalities of supporting their young person through the treatment. In a small minority of cases, caregivers reported young people's behaviour worsened and attributed this to the intervention (and specifically having to think about their trauma(s); although in some of these cases, these behaviours ultimately improved after an initial decline). In some cases, particularly young people in residential care, professionals felt unable to support this decline in behaviour/symptoms. Some short‐lived initial worsening of symptoms may be in response to reducing the use of avoidant coping strategies and engaging in emotional or cognitive processing of the trauma. Future research would be helpfully directed towards understanding how best to ensure young people can remain engaged in treatment where there may be initial increases in concerns or challenging behaviour, and how foster carers and other professionals can be adequately supported over this phase.

### Challenges for a future trial

#### Recruitment, randomization, and retention

A number of challenges were encountered relating to recruitment and randomization. Although most social workers screened at least one young person on their caseload, overall screening rates were low, inconsistent, and often required significant prompting from the research team (raising issues of scalability/sustainability). The pace and overall rate of screening made it difficult to ensure enough young people were identified to randomize to a group. Similar challenges have also been reported when trying to recruit and randomize for group‐based interventions with refugee youth (Rondung et al., 2022). With input from our independent steering committee, randomization was ultimately dropped. That is, we deemed it unfeasible to randomize young people in care to a group intervention within a single local authority. Whilst mental health screening by social workers is possible, as a sole approach it is unlikely to be an effective strategy of identifying young people who may benefit from this type of intervention. Similar issues have been identified by other research groups, highlighting structural and cultural barriers to implementation of routine mental health screening (Devaney et al., 2023). Any future trial would need to explore other options for trial design, such as a waitlist control or randomization between local authorities, rather than within, and consider multiple inroads for screening (e.g., at the social care, foster carer, and mental health level).

Of note, the intervention being targeted (i.e., for those screened as experiencing symptoms, as in the current study) or universal (i.e., provided to a population based on trauma exposure, not symptoms) was not shown to moderate treatment effects in a meta‐analysis of group interventions for child PTSD (Davis et al., 2023). Thus, a future trial may consider removing initial screening altogether, and rather providing the intervention to any young people in care who wish to attend, given the well‐documented high rates of trauma exposure and mental health needs in this group (Bronsard et al., 2016).

Retention rates were generally acceptable – most young people who started the intervention completed at least three of the five sessions, with the majority finishing all five. Almost 80% of recruited young people completed at least one follow‐up assessment (i.e., either post‐treatment or 3‐month follow‐up). Where follow‐ups were completed, the main symptom measures were well‐completed. However, lower priority measures (those later in the questionnaire pack) were less‐well completed (~75% of those who participated in the follow‐up, which would be 44% of the baseline sample). The PTSD diagnostic interview, which required a scheduled video or in‐person meeting, had lower completion rates at all time points. Any future trial would need to carefully consider participant questionnaire burden. This includes considering the centrality of understanding the presence or absence of diagnosed PTSD (vs. symptom severity). Whilst diagnosis has traditionally been gold‐standard in RCTs, in practice, UK mental health services rarely use full‐scale diagnostic interviews, whilst there is also growing evidence that young people in care underreport their symptoms (Tarren‐Sweeney, 2019), suggesting caution around pre‐specified cut‐offs. Of concern was the lack of follow‐up assessments completed on young people who disengaged from the treatment. Any future trial would need to ensure adequate resources to develop and test strategies to maintain engagement with young people who drop‐out of treatment.

#### The absence of stepped‐care

TRT is a low‐intensity intervention. It provides a potentially highly scalable option for addressing a large number of young people in need, where high‐intensity treatments alone are unlikely to feasibly meet need. However, high‐intensity treatments (i.e., one‐to‐one trauma‐focused CBT) remain the best evidenced and most effective treatments for young people with PTSD, including those exposed to abuse or maltreatment (Davis et al., 2023; Hoppen et al., 2023; Mavranezouli et al., 2020). Qualitative reports and anecdotal evidence from therapists suggested young people who engaged in the intervention often wanted further support following the five sessions. However, there are currently major capacity issues within UK CAMHS (Children's Commissioner for England, 2023), and there was no option for young people to access best‐evidenced care, even when requested. There are important ethical considerations when delivering low‐intensity interventions to complex groups of young people, when best‐evidenced care is not available. It is too soon to draw conclusions on the appropriateness of this, but any future trial would need to carefully monitor for adverse events and access to further support when indicated. Low‐intensity interventions such as TRT, may best fit within a stepped‐care approach that would allow young people to access best‐evidenced high‐intensity interventions where needed.

### Limitations and considerations

This was a feasibility and acceptability study of a low‐intensity scalable intervention delivered to young people in care experiencing elevated PTSD symptoms (along with other complexities and comorbidities). It answered many important key considerations required for a fully‐powered trial. However, all findings should be interpreted in light of this being a small‐scale feasibility trial with no randomization.

The COVID‐19 pandemic meant we ultimately delivered a number of groups online. Although this allowed for interesting extended learning of delivery format, the intervention was not designed to be delivered online and our findings suggested the online delivery was not as helpful and may have facilitated avoidant coping. It also hindered the benefits of shared learning and experience. If future trails of TRT, or other group interventions, proceeded with online delivery, they should be more fully modified and co‐developed with young people to ensure the online translation does not dilute the benefits of the treatment.

We were ultimately unable to assess procedural questions around randomization – although the ability to randomize was itself a key feasibility question. Nevertheless, we were unable to obtain data on retention rates for a control group or what ‘care as usual’ would look like for a control group. That said, we did gain some insight into these issues via data collected from the intervention participants. Our lack of follow‐up for those who dropped‐out of the intervention also strongly suggests a future trial would need to be appropriately resourced to allow significant efforts to follow‐up any young person who was not actively engaged in the treatment. Finally, children's social care is under‐funded and over‐stretched, with high staff turnover. This poses challenges for research and we were unable to collect data on how many young people social workers were approaching for initial screening, meaning we do not know how many young people declined initial screening.

### Summary

With some careful considerations and modification to the trial design, TRT could potentially be assessed via a fully‐powered multi‐site trial. However, there are a number of significant considerations before any such trial. Key logistical considerations would include: whether the initial screening stage is needed (Davis et al., 2023); the type of trial design (e.g., avoiding randomization within a single site and instead using a cluster trial design); how to retain participants who drop‐out of the intervention (or who are in a potential control group); how to ensure adequate comparative data; and how to ensure caregivers and professionals (foster carers, key workers, social workers) are appropriately trained to support young people through the intervention. However, central to considering a future trial is that low‐intensity options may be inappropriate for some young people where follow‐on high intensity best‐evidenced support (via stepped‐care) is not available. Well‐documented capacity issues within the NHS (Children's Commissioner for England, 2023; NHS Confederation, 2022) mean many services for young people in care (and for young people in general) do not offer best‐evidenced interventions. The field does not yet provide the necessary evidence for services to empirically determine which young people could benefit from a low‐intensity intervention and which may need further support. Any future trial might be best place to evaluate a stepped‐care model as part of assessing TRT, and would need to carefully monitor for potential adverse outcomes.

## AUTHOR CONTRIBUTIONS

**Rebecca S. Davis:** Data curation; formal analysis; visualization; writing – original draft; project administration. **John Devaney:** Conceptualization; writing – review and editing; methodology. **Sarah L. Halligan:** Conceptualization; writing – review and editing; methodology; supervision. **Richard Meiser‐Stedman:** Resources; methodology; writing – review and editing; conceptualization; supervision. **Paula Oliveira:** Formal analysis; visualization; writing – original draft; writing – review and editing. **Patrick Smith:** Conceptualization; methodology; supervision; writing – review and editing; resources. **Paul Stallard:** Conceptualization; writing – review and editing; supervision; methodology. **Rebecca Kandiyali:** Conceptualization; writing – review and editing; methodology. **Alice Phillips:** Data curation; formal analysis; writing – review and editing. **Aalia John:** Writing – review and editing; data curation; formal analysis. **Rachel M. Hiller:** Conceptualization; formal analysis; visualization; writing – original draft; methodology; supervision; project administration; funding acquisition.

## FUNDING INFORMATION

This project was funded by a National Institute for Health and Care Research (NIHR), Research for Patients Benefit (RfPB) grant, awarded to RMH. The views expressed here do not necessarily reflect the views of the NIHR.

## CONFLICT OF INTEREST STATEMENT

None.

## Data Availability

The data that support the findings of this study are available on request from the corresponding author. The data are not publicly available due to privacy or ethical restrictions.
